# Monitoring changes in the Gene Ontology and their impact on genomic data analysis

**DOI:** 10.1093/gigascience/giy103

**Published:** 2018-08-09

**Authors:** Matthew Jacobson, Adriana Estela Sedeño-Cortés, Paul Pavlidis

**Affiliations:** 1 Michael Smith Laboratories, 177 Michael Smith Laboratories, 2185 East Mall, University of British Columbia, Vancouver BC V6T1Z4; 2 Department of Psychiatry, 177 Michael Smith Laboratories, 2185 East Mall, University of British Columbia, Vancouver BC V6T1Z4; 3 Graduate Program in Bioinformatics, 177 Michael Smith Laboratories, 2185 East Mall, University of British Columbia, Vancouver BC V6T1Z4

**Keywords:** genetics, genomics, ontologies, gene function, bioinformatics

## Abstract

**Background:**

The Gene Ontology (GO) is one of the most widely used resources in molecular and cellular biology, largely through the use of “enrichment analysis.” To facilitate informed use of GO, we present GOtrack (https://gotrack.msl.ubc.ca), which provides access to historical records and trends in the GO and GO annotations.

**Findings:**

GOtrack gives users access to gene- and term-level information on annotations for nine model organisms as well as an interactive tool that measures the stability of enrichment results over time for user-provided “hit lists” of genes. To document the effects of GO evolution on enrichment, we analyzed more than 2,500 published hit lists of human genes (most older than 9 years ); 53% of hit lists were considered to yield significantly stable enrichment results.

**Conclusions:**

Because stability is far from assured for any individual hit list, GOtrack can lead to more informed and cautious application of GO to genomics research.

## Background

The Gene Ontology (GO) has been widely adopted by computational and experimental biologists, and Gene Ontology annotation (GOA) of genes is one of the most prominent descriptive features of major genome databases. The original paper describing GO [1] is among the most cited papers in the biomedical literature (more than 14,000 citations, Clarviate Analytics Web of Science, accessed January 2018). The popularity of GO is in large part due to the challenge of interpreting data generated from high-throughput technologies such as gene expression profiling.

In a typical simple setting, researchers contrast a genome-wide feature (e.g., gene expression levels or genetic association) in two experimental conditions and generate a list of genes, either ranked across the whole genome or in the form of a “hit list” of selected candidates. Another way such lists can be generated is by clustering, such as using protein interaction networks or coexpression, or by selecting genes harboring potentially pathogenic variants in cohort-based genome sequencing. To help extract biological meaning from those rankings and hit lists, it is now standard practice to use GO annotations in an “enrichment” framework.

The widespread use of these methods suggests it is important that users understand their underpinnings. However, despite the importance of GO, many users likely have little understanding of how it is developed, despite some effort on the part of the GO Consortium (GOC) to disseminate such information [2–4]. An important feature of GO is that it changes over time, as curation is performed. This has potentially major implications for the utility and interpretation of GO/GOA, but there is currently no means for users of GO to easily see this for themselves. Our goal is to help fill this gap and provide some insight into the actual impact of changes on data analysis.

The structure, content, and curation of GO/GOA are the essential backdrop for the work we present, so we review it briefly. It is important to distinguish the GO itself (the ontology) from the annotations (GOA), which connect genes to terms in GO. GO is organized into three sub-ontologies, representing biological processes, molecular functions, and cellular components. Collectively these currently encompass more than 40,000 concepts, arranged in a directed acyclic graph (like a tree, but with the potential for multiple paths from any leaf to the root).

Curation is managed through the GOC, in which member organizations, such as model organism database curation teams, provide annotations to a central repository. Genes may be associated with terms in the ontology using either manual curation (associated with a specific reference to the literature or based on a computational analysis reviewed by a curator) or “automatic” annotations that are not reviewed by curators. The different types of associations are represented by evidence codes, e.g., the automatic annotations receive the code “IEA” (“inferred from electronic annotation”).

Annotations created by the curation process are referred to as “direct annotations” because they explicitly associate a GO term with a gene. Genes are also associated with terms indirectly via the graph structure of GO, referred to as inference. Thus, a gene that is directly annotated with the term “protein tyrosine kinase” is also implicitly annotated with the term “protein kinase” because that term is a parent term of “protein tyrosine kinase.” When the operation of propagating direct annotations through the GO hierarchies is completed (“transitive closure” in graph theory terminology), the number of annotations available greatly increases, albeit at a range of granularities. These “indirect annotations” (also referred to as “inferred” or “propagated”) are as valid as direct annotations because GO enforces a “true path” rule [5]. In most analyses, it is important to use propagated annotations (the combination of direct and inferred annotations) [6].

Assessments of GO/GOA have recently turned to considerations of changes over time. For example, we quantified the effect that annotations have on the apparent (annotated) function of genes, showing that, on average, changes over short periods (months) are minor, but changes over longer periods are much more substantial [7]. This and other work has shown that GO enrichment results may not be stable over time. However, the effects of changes are not likely to be uniform across datasets nor easily predictable. Indeed, previous studies have been either anecdotal (considering a single or just a few examples [8–11]), with the largest study analyzing around 100 [12], or yielded mixed findings. Groß et al. (2012) found that enrichment results were stable based on analysis of two hit lists. Alam-Faruqe et al. considered changes in results to be improvements due to focused curation, based on analysis of two datasets. Others have emphasized instability [11,12] or reported mixed impacts [9]. Given this variety of results and interpretations, there is clearly a need for researchers to assess the stability of their own specific enrichment results.

Here, we report the development and application of a database (GOtrack) that contains historical information on GO going back to the early 2000s for human and major model organisms. The GOtrack web site enables quick exploration of GO and GOAs over time and evaluation of how changes impact interpretation of analyses derived from GOA. Using the data in GOtrack, we present several analyses of trends in GOAs, complementing earlier work. We performed a large-scale analysis of enrichment analysis results over time, using a large corpus of more than 2,500 “hit lists.” We confirm that GO enrichment analysis results can change over time. However, many were stable by objective measures even over time spans of greater than 10 years. It is our hope that GOtrack will enable more critical use of GO by biologists and computational researchers.

## Findings

### Construction and overview of GOtrack

**Figure 1:**
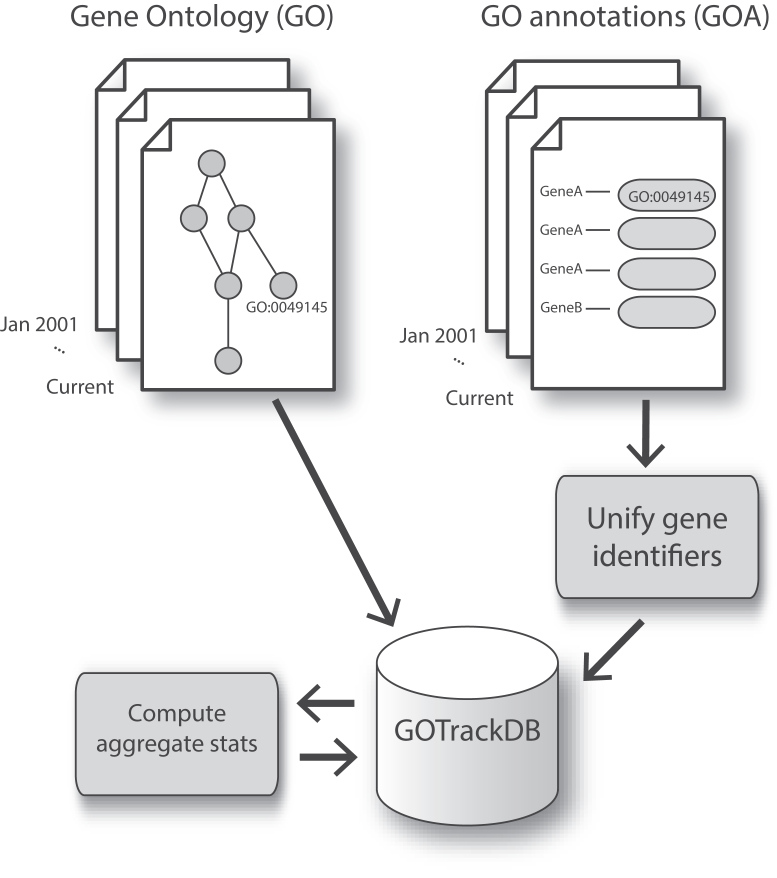
Overview of approach in constructing GOtrack. GO terms and GOAs were obtained and matched by date, and gene identifiers were harmonized. Precomputed summary and aggregate statistics supplement the fine-grained information stored in the databases.

We used data representing ontologies and annotations for nine organisms, dating as far back as 2001. Annotation data were not available for all organisms for all dates, with complete data for all nine organisms from 15 November 2011 onward. In total, the data encompasses 206 monthly versions of GO and 1,545 species-specific monthly editions of GOA, yielding 206,894,446 GO annotations (as of January 2018). Our overall procedures are outlined in Fig. 1 (see Methods; additional information is available on the GOtrack web site). The resulting database is complex and rich, with extensive information available at the gene or GO term level. While the web interface is the most complete and detailed way to interact with the data, we also offer a RESTful API to enable programmatic access to the data. Via this API, users can download GOAs for a taxon, as well as GO, for any selected point in time. GOtrack does not contain all information on GO/GOA and thus complements other resources such as QuickGO [13] and AmiGO [14].

The GOtrack web interface offers views of history at the gene level and at the GO term level. A third view provides a “global overview” of trends according to a variety of statistics. Finally, we offer a web tool to track changes in GO enrichment results over time. Here, we provide only a high-level overview of basic functionality. Readers are invited to explore the web interface for more information.

**Figure 2:**
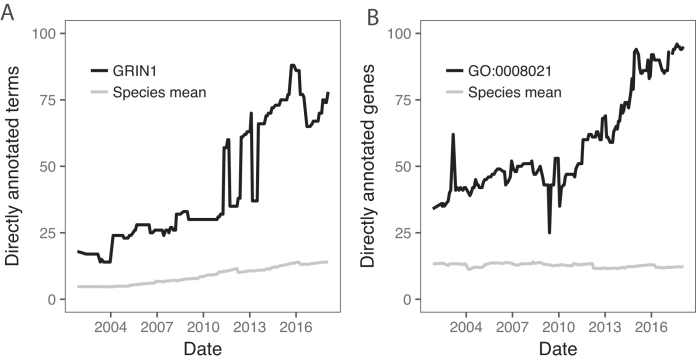
Examples of information provided by GOtrack for genes and terms. **(A)** Number of terms directly annotated to the human gene GRIN1. Large drops and rises are observed superimposed over a general gradual increase in annotation since 2002 (black). In this example, the large shifts are not accompanied by corresponding shifts in the species average (gray). **(B)** Number of human genes directly annotated with the term “synaptic vesicle” (GO:0 008021) over time, again showing transient drops and rises. Data from GOtrack were replotted for presentation. For corresponding screenshots, see Supplementary Fig. S1.

Figure 2A shows an example of one type of data offered in the gene view, for the human gene GRIN1 (glutamate ionotropic receptor NMDA type subunit 1; [15] and Supplementary Fig. S1A). The plot shows the number of GO terms directly annotated to the gene, with the mean of all genes from the same organism plotted for comparison. GRIN1 is consistently more highly annotated than the average, and its trajectory is typical in that annotations rise over time, interrupted by drops and recoveries. In general, such changes can be due to either annotation curation—addition or removal of terms annotated to the genes—or changes in the structure or content of the GO itself, such as addition of terms or relations. The GOtrack interface also allows users to inspect changes in the use of evidence codes used to support an annotation and to directly compare annotations for a gene at up to four time points.

To help users interpret the changes in number of terms over time, we provide additional plots of statistics derived from the annotations. The first of these is of multifunctionality [16,17], which is related to the number of terms annotated to a gene, with a weighting to account for term specificity (where specificity is defined by how many genes are annotated with the term; see [17] for details). This more precisely captures how heavily annotated the gene is relative to other genes. The second derived statistic is semantic similarity. As time passes, changes in annotations can cause a gene to change “functional identity” [7]. To quantify this effect, we plot the Jaccard index between the annotations in the current edition to each previous edition. These and other plots and tables are presented on the web page for each gene.

The term-level view provides information on how a GO term has changed over time. This includes how many genes were annotated to it either in total (Fig. 2B) or broken down by evidence type (e.g., [18] and Supplementary Fig. S1B) as well as changes in the GO structure that impact the term's relationships. Finally, the Global Trends page [19] shows species-level summaries of the numbers of annotated genes, genes annotated per term, annotations per gene, and the size of GO itself.

### Long-term trends in GOA

In this section we present some analysis of the data in GOtrack, focusing on annotations (rather than GO itself). As noted, genes vary strongly in how highly annotated they are, due to varying degrees of experimental and curation attention paid to the gene as well as potentially true biological differences in multifunctionality [17]. Previously we reported that this bias tends to persist, i.e., genes that are relatively highly annotated tend to stay that way [7]. We confirmed this is still the case five years later. For example, if we rank genes by how many direct annotations they have, the ranking at the earliest time point is correlated with the ranking at the latest time point (human: Spearman rank correlation 0.52; mouse: 0.43; Arabidopsis: 0.53). Thus, we confirm that genes are not just unequal in their annotation; we confirm that this inequality is stable over long periods.

**Figure 3:**
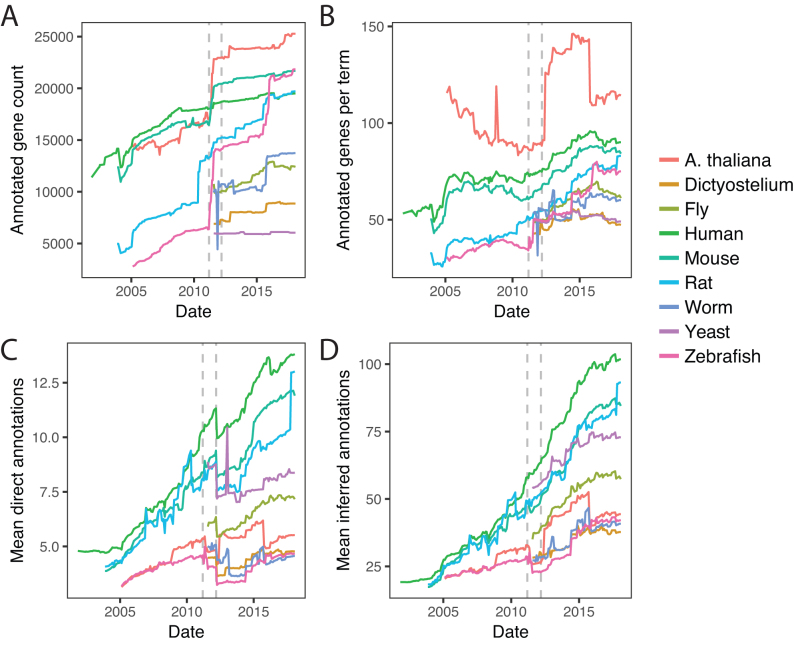
Trends in taxon-wide annotation statistics. **(A)** Number of annotated genes. **(B)** Mean annotations per term (inferred + direct). **(C)** Mean number of direct annotations per gene. **(D)** Mean number of inferred (including direct) annotations per gene. Times of prominent discontinuities affecting multiple species in A and C are marked by dashed gray lines in all four panels.

The jumps seen in individual genes (e.g., Fig. 2A) are not all independent events, as the course of the species-wide averages also has discontinuities (Fig. 2A, gray). This is also apparent in a principal components analysis of the direct count matrix (Supplementary Results, Supplementary Fig. S2). We investigated this more completely in all nine GOtrack organisms at the level of total gene coverage (Fig. 3A), genes annotated per term (Fig. 3B), direct annotations per gene (Fig. 3C), and inferred annotations per gene (Fig. 3D). This reveals that large jumps and drops are sometimes simultaneously observed in multiple, or even all, species. One such notable event was a rapid increase in the number of annotated genes starting March 2011 for Arabidopsis, mouse, and zebrafish (Fig. 3A). Another dramatic event was a large drop in the mean number of direct annotations per gene in March 2012 for all species (Fig. 3C). The jump is not visible in the plots for indirect annotations (Fig. 3D). This would be consistent with a large-scale purging of redundant annotations (rejecting higher-level terms that are inferable from more specific terms). Other jumps are species specific, such as the large increase in Arabidopsis genes annotated per term in early 2012, followed by a large drop in late 2015 (Fig. 3B).

At the gene level, large shifts in the numbers of annotations can be due to removal and replacement of annotations for the same term—a phenomenon we call “annotation churn.” For example, for the human gene ACTC1 (Actin, Alpha, Cardiac Muscle 1) [20], there is a pronounced rise in annotations in mid-2007, with a one-month dip in May 2008 (see screenshots in Supplementary Fig. S3). GOtrack makes it easy to drill down into details. By examining the tabular results (Supplementary Fig. S3A), it is found that one of the terms that was deleted during the dip was “apoptosis” (GO:0 006915). Viewing the annotation history for that term on the gene, we see that the term was repeatedly added and removed (in 2007-2008), with the evidence code “IEA.” In June 2008 the term was annotated to the gene with a higher-grade curator-reviewed evidence code (ISS), where it remained (the term was also renamed to “apoptotic process”) until it was removed again in December 2017 (Supplementary Fig. S3B).

### Tracking enrichment results

In addition to the exploratory aspects described so far, the other major component of the GOtrack system is an analysis tool that performs enrichment analysis at multiple time points ([21]; Supplementary Fig. S4). The key idea is to observe whether an enrichment result is stable relative to a given point in time. The main input provided by the user is a “hit list” of genes. The output includes plots and detailed tables to help interpret the results and judge whether the results change over time. This includes direct comparisons of “before and after” sets of enriched terms. The measures we use for this comparison are discussed in the next section and in the Methods section. In addition to these statistics that summarize the overall stability of the results, the web interface provides term-level stability measures. This makes it easy to see whether a term has been consistently “significant” over past editions.

The enrichment tool has some limitations. We use a simple overrepresentation method (as do many tools, including the popular DAVID [22]). Also, the “background” set of genes is not settable by the user; it is the set of all genes annotated at the particular time point. Because GOtrack provides downloads of GO and GOA for any date, users can confirm findings with the software of their choice, provided it allows user-provided GO and GOA as inputs (such as ErmineJ, [16], whose annotation input format is directly supported).

### Evaluating the stability of enrichment results

We hypothesized that changes in GO/GOA over time could cause changes in enrichment results to such an extent that they would be effectively unrecognizable and lead to a different interpretation of the results. As described in the introduction, previous studies of this question yielded somewhat mixed results on small numbers of test hit lists. In our approach to this question, we used a corpus of gene lists from the Molecular Signatures Database (MSigDB) [23]. These are divided into two groups (after filtering, see the Methods section): 1,327 curated “canonical pathways” (CPs) and 2,573 “chemical and genetic perturbations” (CGPs). The latter correspond to published hit lists of the type usually investigated with enrichment analysis. We took advantage of the fact that each CGP hit list is associated with a publication, allowing the opportunity to see if the enrichment results obtained around the time of publication would have changed in the interim. We predicted the CP lists of established pathways would be more stable compared to the experimental CGP hit lists. The limitation of the MSigDB corpus is that most of the publications are not very recent (median 11 years; range, 0.4–16, 90% are >9.2 years old) and we have done little investigation of short-term stability.

For each hit list or pathway, we compute results of an enrichment analysis as it would have appeared at the GO/GOA edition nearest to the source publication date (see Methods section for details). We then repeated the enrichment analysis using the most current GO/GOA edition (January 2018). This results in a range of timespans to have passed following publication. For the CP set, which do not all have an associated date, we computed results for the most recent GO/GOA edition and the earliest date available (January 2001). We used this extreme date for comparison because we expected the CP set to have greater stability, so comparing to the earliest date is the “worst-case scenario” for comparing to the experimentally derived CGP sets.

**Figure 4:**
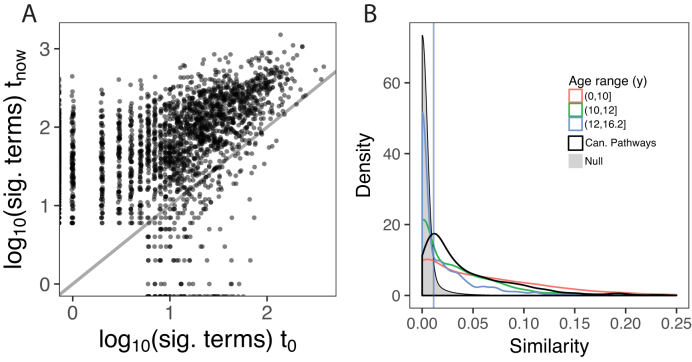
Stability analysis of 2,573 published hit lists. **(A)** Change in number of significant GO terms. Each point is one CGP hit list. Points are jittered to reduce overplotting. **(B)** Similarity of enrichment results, using the complete Jaccard index. The CGP hit lists are binned into most recent (orange), old (green), and oldest (blue). The distribution for the CPs is in black. The blue vertical line indicates the 95%ile of the null.

Our first key observation is that on average for the CGP hit lists, the number of significant terms goes up dramatically (from 21 ± 32 terms to 110 ± 136 terms, mean ± standard deviation; *P* < 10^−15^, Wilcoxon rank sum test). The values are highly correlated (Fig. 4A); hit lists that had few significant terms at the time of publication (henceforth t_0_) had relatively few at the most recent time point (t_now_) (rank correlation 0.54). These results also held for the CPs (growing from 37 ± 59 to 246 ± 216 terms, correlation 0.57). It is likely that these increases are not just due to increased annotation but also to the growth of GO to more than 47,000 terms of increasing granularity.

The explosion in the number of significant terms is an obvious form of instability. Of course, what matters more is whether the enriched terms resemble each other at t_now_ compared to t_0_. To evaluate this, we did direct comparisons of the enriched terms associated with each hit list (at t_0_ and t_now_), using the Jaccard index (see Methods section and Supplement). The Jaccard index was calibrated using a null distribution created by comparing pairs of unrelated hit lists (see Methods section). To simplify the analysis, we binned the CGP hit lists by age into three groups of similar numbers of hit lists: up to 10 years, 10–12 years, and 12–16 years.

The results are shown in Fig. 4B. Overall, 53% of the CGP hit lists had results that were more similar than 95% of the null trials. This fraction is much higher for relatively recent lists (71%, N = 640) and lower for the older lists (55% for the middle tranche, N = 960, and 38% for the oldest, N = 973; Fig. 4B). In comparison, 75% of the CPs remained above this threshold, despite most of the comparisons being done to the earliest possible time point. The overall rank correlation (unbinned) between stability and age is −0.34 (CGP; −0.39 for CPs). This demonstrates that it is possible for results to maintain a substantial degree of similarity over periods of greater than 15 years and that, in general, drift in the semantic content of enrichment results is very substantial after 12–16 years and is substantial but less striking at shorter time spans (<10 years). In the Supplement we present examples of hit lists yielding high and low stability (Supplementary Results and Supplementary Files).

A notable feature of the data shown in Fig. 4B is that very low values of the complete Jaccard index were statistically significant. This shows the importance of using a null distribution to calibrate the scores but clearly leaves something to be desired as a Jaccard index of 0.01 seems negligible. However, this effect is due, in large part, to the increase in the number of terms over time (Fig. 4A), guaranteeing that the Jaccard index will drop. In attempts to explore this further, we tested six variants on the Jaccard index (see Supplement). While some of the alternatives have scales that are more intuitively matching expectations of what “stable” would represent on a scale of 0–1 (e.g., with 95%ile of the null equal to 0.41), the findings are qualitatively similar to the complete Jaccard index (data for two additional measures are shown in Supplementary Fig. S5). Several of these alternative measures are implemented on the GOtrack web site. These measures are discussed further in the Supplement in the context of examples, along with discussion of the subjective nature of comparing enrichment results in an exploratory analysis.

We looked for factors that might contribute to stability. For the CGP hit lists, the number of genes in a hit list was not strongly predictive of Jaccard stability (rank correlation 0.18). It was only modestly correlated with the mean number of directly annotated terms (−0.12) or mean multifunctionality of the genes in the hit list (−0.09). There were more obvious trends for the CP lists, which have higher stability than the CGP lists on average, despite the (artificially) long time passed between t_0_ and t_now_ (more than 12 years; Fig. 4B). The number of direct annotations per CP is higher (36 vs. 25.4 for CGP). However, this does not appear to explain the overall higher stability of the CP lists, because we get the same result for the subset of CP that has <35 mean direct annotations (mean of 22.9; correlation is −0.48; overall correlation is −0.46). Thus, hit lists that have more highly annotated genes have a tendency to be less stable. However, given these low correlations (−0.12 for the CGP set) and without further insight, it appears to be difficult to predict (even in hindsight) which hit lists will yield stable results.

## Discussion

In this work we present GOtrack, which to our knowledge is the only resource available that allows easy access to historical data on GO/GOA and the only one that allows inspection of the effects of changes over time on enrichment result stability. Our analyses further highlight the necessity for users of GO/GOA to be cautious in their interpretation of any GOA and to temper whatever trust they have in GO enrichment results.

Our evaluation of the stability of enrichment results differs in several important ways from earlier efforts. First, we matched GO and GOA for each time point (rather than fixing either GO or GOA while varying the other), which we feel is more realistic. We also analyzed a much larger number of hit lists (>2,500 vs. a maximum of ∼100 [12]) and considered time of publication to ensure comparisons were also realistic. Perhaps most importantly, we used a null distribution to calibrate the similarity measures, providing improved objective measures of what qualifies as stability. Overall, our results are more optimistic about stability than those of Tomczak et al. (2018). Regardless, we concur with previous reports that changes in GO/GOA can make a substantial difference in results. However, because of the high degree of variability and difficulty in finding fully satisfying quantitative measures that are often interpreted subjectively (see Supplement for discussion), our recommendation is that users of GO should judge for themselves by using GOtrack. Researchers who are reporting enrichment analyses can check which terms have been stable (e.g., over the last five years). This provides a principled way to help narrow down complex enrichment results, which is a problem that many users of enrichment analyses struggle with.

An obvious limitation is that GOtrack cannot see into the future. While the stability of any particular GO enrichment result might be high or low when looking back in time, it is generally impossible to know whether it will remain stable because knowledge of biology as represented in GO/GOA is a work in progress. Indeed, we found it is difficult to predict which hit list would give stable results. The strongest clue we could identify is how well annotated the genes in the hit list are. Hit lists with highly annotated genes (mean direct annotation count) tend to be less stable. We speculate that this is because highly annotated genes have more changes to their annotations, which can drive shifts in enrichment results. However, we have yet to explore this further, and, in any case, the relationship is not strong enough to be usefully predictive. In addition, we did not assess other possible factors influencing stability such as evidence codes [24], which is a topic we leave for future research.

GOtrack currently has some limitations. The enrichment tool uses a simple method and does not implement algorithms to asses multifunctionality biases [16]. Our data on GO/GOA are not complete. We did not import all of the fields from GOA files, the most useful of which for analysis purposes might be the annotation source. Finally, the recently added concept of annotation extensions [25], which provide context for an annotation (e.g., a cell type), are not handled by GOtrack.

## Conclusions

The evolving and incomplete nature of GO/GOA has always been inherent and is well understood by the GO community, but it is seemingly less appreciated more broadly. For example, the extremely popular enrichment tool DAVID ( more than 32,000 citations as of May 2018 [26]) did not update its GOAs for nearly seven years, an eon in GO history (and, at the time of this writing, DAVID had not been updated for nearly two years [27]). We find it interesting that there was no massive outcry in response to the use of such out-of-date GOAs, suggesting either ignorance or apathy. While it might seem obvious that one would always want to use the latest GOAs, this can be questioned. GO/GOA can change dramatically in a seesaw fashion over a period of months, suggesting that not all changes are improvements. Furthermore, we report a strong tendency for hit lists to yield ever more significant terms over time (Fig. 4A), and it is not clear that this comes with any increase in useful information. It could be that using GO/GOA from an earlier, simpler era might be beneficial for enrichment analyses (using a GO slim [28] may approximate this concept). While we may not be able to settle that question here, it is clear that whatever version of GO/GOA is used, it cannot be treated as a gold standard. Enrichment analysis should be considered exploratory and never be used as a primary finding [29]. Computational researchers should also be cautious in using GO/GOA as an optimization target when developing and evaluating algorithms, especially since changes over time are not the only concern [7,17].

GOtrack should be a valuable resource for biologists to gain a greater understanding of where GOAs come from and how they change over time, as well as their impact on the major use case for GO/GOA enrichment analysis. Our analysis of the data in GOtrack also revealed a number of interesting features, and it is likely that deeper analyses can be used to gain more insight into patterns of curation that might influence future efforts.

## Methods

### Gene Ontology

Historical GO files were retrieved from [30], specifically, dates between 1/1/2001 and 1/3/2004 were obtained from separate process.ontology.<date>.gz, function.ontology.<date>.gz, and component.ontology.<date>.gz files and subsequently combined. Dates between 1/4/2004 and 1/10/2006 were obtained from gene_ontology.obo.<date>.gz. Dates after 2006-10-01 were obtained from gene_ontology_edit.obo.<date>.gz. These files exclude relationships that cross the three GO aspects, and we restrict our analysis to IS_A and PART_OF relationships only.

### GOAs

Historical species-specific annotation files were retrieved from [31], specifically, dates between 2/11/2001 and 9/5/2016 and were obtained from gene_association.goa_<species>.<edition>.gz. Dates after 9/5/2016 were obtained from a combination of goa_<species>.gpi.<edition>.gz and goa_<species>.gpa.<edition>.gz files. Mapping of historical annotations to a release of the GO was done by selecting the ontology with the closest release date before that of the annotation file. Annotations were propagated up the GO graph as per the “true path rule” [5]. To convert release editions to dates prior to edition 135 (July 2014,) the release number of the file is compared to the dates given on the GOA news site [32]. For edition 135 onward, we use the date provided in the files. We note that there are some gaps in the available data, especially at early time points. For example, we lack data for human for September 2002 and October 2002. In addition, the spacing of dates is not uniform; while the median inter-edition gap is 28 days, there are a few gaps that are smaller (minimum 13 days) or correspondingly larger (e.g., 40 days).

### Mapping of gene identifiers over time

Gene product annotations are tracked historically using their associated UniProt accession number(s) [33]. Each gene product in UniProt has a unique primary accession, called the “primary (citable) accession number.” In addition to this, a gene product may also have secondary accession numbers that could have been created historically from merges and/or splits. During a merge, the first accession is retained as the primary while all others become secondary. During a split, a new primary accession is created for all products involved while their original accessions are retained as secondary. An accession is only deleted when its corresponding entry has been removed from UniProt. The mapping of primary to secondary accessions is retrieved from [34]. This mapping allows us to find the current primary accession of a historical annotation.

### Enrichment analysis

GOtrack implements overrepresentation analysis using the hypergeometric distribution [16]. The background is the set of all annotated genes (for the time point being analyzed). For analyses presented here, terms with between 20 and 200 genes were included, and only Biological Process terms were considered. The false discovery rate was controlled at 5% using the method of Benjamini and Hochberg [35]. The GOtrack enrichment tool allows these parameters to be varied by the user.

### Data analysis

Many of the analyses described are based on files available via the GOtrack web site [36], including the “summary” files by edition, terms, and genes. Analyses were conducted with custom scripts written in R [37,38] and Python. Correlations are Spearman rank correlations except where indicated otherwise.

### Analysis of MSigDB hit lists

The MSigDB C2 collection [23] was downloaded from [39]. This corpus is divided into a set of CPs and CGPs. For the CGP hit lists, the publication associated with each hit list was extracted, and the date of publication (t_0_) was used to identify the nearest matching version of GO/GOA in GOtrack. Each hit list was analyzed for enrichment as described above, for t_0_, and a recent comparison time point (January 2018, t_now_). We analyzed 2,573 CGP hit lists that yielded at least five significant terms at either (or both) t_0_ or the comparison time point. CP lists (n = 1,327 after filtering) were treated the same way, except t_0_ was fixed at 21 November 2005 (the mean date for the CGP lists).

To compare two sets of enrichment results, we explored several measures (see Supplement) but focus on a standard Jaccard index: $| {E0 \cap E1| / |E0 \cup E1} |$, where E0 and E1 are the sets of all significantly enriched GO terms for the same input hit list at two time points (“complete Jaccard”). The primary alternative measure we examined was a modified Jaccard that examines only the top five terms plus their inferred parent terms (“top-term-parents Jaccard”), similar to the measure proposed by [40]. See the Supplement for details and discussion.

To generate a null distribution, we compare enrichment results from pairs of randomly selected hit lists (i.e., coming from different publications). Instead of comparing a hit list's results for t_0_ to t_now_, the data are permuted so t_0_ of one hit list is compared to t_now_ for a randomly selected hit list (with the same constraint that at least one of them must have five or more significant GO terms). We analyzed 1,000 such permutations of the data and pooled them to generate the null distribution. This is an appropriate null because if two enrichment results from the same experiment (at two different time points) are less similar than what would be expected for two randomly picked independent experiments, we can say that the enrichment results are no longer similar according to the measure. This null also inherently addresses the tendency of some GO terms to recur more frequently than others in independent enrichment analyses [16].

### Implementation

GOtrack is implemented in Java and JavaScript and uses the PrimeFaces framework, with a MySQL database. The open source Highcharts (highcharts.com) visualization library is used for plotting. The data in GOtrack are automatically updated monthly. Because of the lag in when data are available from GOC, data for a given date appear in GOtrack up to 2 months after the stamped date.

## Supplementary Material

GIGA-D-18-00204_R1.pdf

Response_to_Reviewer_Comments_Original_Submission.pdf

Reviewer_1_Report_(Original_Submission) -- Rachael Huntley 7/5/2018 Reviewed

Reviewer_2_Report_(Original_Submission) -- Mark Wass 7/25/2018 Reviewed

Supplemental Files

## Availability of source code and requirements

- **Project name:** GOtrack
- **Project home page:** https://gotrack.msl.ubc.ca/[41]; source code at https://github.com/PavlidisLab/gotrack [42]. Code and all data files needed to reproduce the analyses presented are provided at [43]. An executable workspace is also available [44].
- **Operating system(s):** The web application runs under Linux. The web application works with major desktop operating systems (MacOSX, Windows, Linux).
- **Programming languages:** Java 8, JavaScript, Python.
- **Other requirements:** The web application works with major web browsers.
- **License**: The contents (images, text, data) of the GOtrack web site are released under the Creative Commons BY-SA 2.0 license. The GOtrack source code is open under the Apache 2.0 license.
- RRID:SCR_016399 An archival copy of the code and supporting data are also available via the *GigaScience* GigaDB repository [45].

## Additional files

- Supplementary results and discussion
- Supplementary Figure 1: Screen shots of the gene and term views in GOtrack
- Supplementary Figure 2: Principal components analysis of the direct annotation count matrix
- Supplementary Figure 3: Screen shots showing annotation history tracking for a gene (annotation churn)
- Supplementary Figure 4: Enrichment web interface
- Supplementary Figure 5: Analysis of MSigDB lists using alternative similarity measures
- Supplementary Figure 6: Correlations of stability measures
- Additional Files 1–3: Microsoft Excel spreadsheets providing examples of CGP enrichment results discussed in the supplement. (APPEL_IMATINIB_RESPONSE.enrichment.xlsx, BENPORATH_ES_2.enrichment.xlsx, ONDER_CDH1_SIGNALING_VIA_CTNNB1.enrichment.xlsx):

## Abbreviations

CGP: chemical and genetic perturbation; CP: canonical pathway; GO: Gene Ontology; GOA: Gene Ontology annotation; GOC: Gene Ontology Consortium; GRIN1: glutamate ionotropic receptor NMDA type subunit 1; IEA: inferred from electronic annotation; MSigDB: Molecular Signatures Database .
