# Supplementary material for: Monitoring changes in the Gene Ontology and their impact on genomic data analysis
Source: Gigascience. 2018 Aug 9;7(8):giy103. doi: 10.1093/gigascience/giy103 (PMC6113503; doi:10.1093/gigascience/giy103)
Supplement: GIGA-D-18-00204_R1.pdf [file giy103_giga-d-18-00204_r1.pdf]

GigaScience

Monitoring changes in the Gene Ontology and their impact on genomic data analysis

--Manuscript Draft--

**Manuscript Number:**	GIGA-D-18-00204R1
**Full Title:**	Monitoring changes in the Gene Ontology and their impact on genomic data analysis
**Article Type:**	Technical Note
**Funding Information:**	National Institutes of Health (MH111099) — Dr. Paul Pavlidis<br>Canadian Network for Research and Innovation in Machining Technology, Natural Sciences and Engineering Research Council of Canada (Discovery Grant) — Dr. Paul Pavlidis<br>Canadian Foundation for Innovation infrastructure — Dr. Paul Pavlidis<br>Canadian Institutes of Health Research (Training grant in Bioinformatics) — Ms Adriana Estela Sedeño-Cortés
**Abstract:**	Background: The Gene Ontology (GO) is one of the most widely used resources in molecular and cellular biology, largely through the use of “enrichment analysis”. To facilitate informed use of GO, we present GOtrack (https://gotrack.msl.ubc.ca), which provides access to historical records and trends in the Gene Ontology and GO annotations (GOA).<br><br>Findings: GOtrack gives users access to gene- and term-level information on annotations for nine model organisms as well as an interactive tool that measures the stability of enrichment results over time for user-provided “hit lists” of genes. To document the effects of GO evolution on enrichment, we analyzed over 2500 published hit lists of human genes (most over 9 years old). 53% of hit lists were considered to yield significantly stable enrichment results.<br><br>Conclusions: Because stability is far from assured for any individual hit list, GOtrack can lead to more informed and cautious application of GO to genomics research.
**Corresponding Author:**	Paul Pavlidis, Ph.D.<br>University of British Columbia<br>Vancouver, BC CANADA
**Corresponding Author Secondary Information:**	
**Corresponding Author's Institution:**	University of British Columbia
**Corresponding Author's Secondary Institution:**	
**First Author:**	Matthew Jacobson
**First Author Secondary Information:**	
**Order of Authors:**	Matthew Jacobson<br>Adriana Estela Sedeño-Cortés<br>Paul Pavlidis, Ph.D.
**Order of Authors Secondary Information:**	
**Response to Reviewers:**	Dear Dr. Zauner:<br><br>We greatly appreciate the positive response to the work and the opportunity to submit a revision.<br><br>As there were only minor suggestions, we have not made any major changes. We have hopefully addressed all of the formatting and resource registration

Powered by Editorial Manager® and ProduXion Manager® from Aries Systems Corporation

requirements.We have also created a Code Ocean capsule for the main analyses. At this time, I do not have the GigaDB information to include in the manuscript as I have not yet been contacted by your data curators.

All of the reviewer comments are addressed in the point-by-point, below.

Thank you for your consideration,

Paul Pavlidis PhD

Reviewer reports:

Reviewer #1: The manuscript by Jacobson et al. describes the GOTrack web interface that can be used to monitor the changes in Gene Ontology and its associated annotations over time.This is a novel method that potentially will assist users of the GO and its annotations to interpret their observations and analyses of gene product datasets. The paper is well written and the standard of English is good. As expected from these authors, a good knowledge of the GO and how it is developed and curated is demonstrated.The authors highlight the importance of recognising that GO and its associated annotations are dynamic and therefore any analysis is likely to change over time, a concept which is sadly lacking in many papers that use GO for interpreting results. Additionally, the authors explain that GOTrack can be used, in combination with annotation dataset reports from UniProt-GOA, to focus in on the possible reason for these changes. Therefore, this paper should go a long way to help further educate the users of GO. It has previously been suggested that, when performing enrichment analysis, researchers should look for key, relevant GO terms that have been enriched for their dataset in more than one analysis tool, due to the large variability between tool outputs (https://www.ncbi.nlm.nih.gov/pmc/articles/PMC3235096/), therefore the ability to compare a dataset over time using GOTrack is a very useful addition for assisting researchers to better interpret their datasets in an informed manner. The authors describe the various uses of GOTrack with appropriate examples that are easy to understand and well illustrated with figures. They compare GOTrack with previous evaluations of enrichment result stability and describe the measures they have taken to improve on the earlier studies. They also assist the reader with traceability of datasets by providing the sources of all files they have used. I have only a few minor comments.

Response: Thank you for the supportive comments.

Reviewer: 1. GOTrack currently only enables tracking changes of annotations for protein-coding genes, could the authors indicate whether they have considered including the newer GO annotation datasets that have been made available, i.e. macromolecular complexes and non-coding RNAs? Although there are significantly fewer annotations for these entities, I am aware of no other tools that provide direct enrichment analysis for these gene products, so this would be a very useful addition to consider.

Response: We haven't but will consider it for a future release. We agree that these annotations are much less explored, which is likely partly a function of their smaller number. As they increase there will be more impetus for us and others to build tools that support them.

Reviewer: 2. In Supp. Fig. 3A the authors show a comparison of 4 editions, but on the website it explains only how to compare two. I discovered by trial and error that you can add more editions by using <shift> + <command> + <click>, so this explanation should be added to the website.

Response: It is documented in the "?" tooltip in the upper left-hand corner of the plot. But your difficulty in noticing this led us to change the text to document the command-shift-click combination by default.

Reviewer: 3. Page 9 Line 21: remove 1st "the" from "because the we get the same

	result"<br><br>Response: We have corrected this error.<br><br>Reviewer: 4. Page 10 Line 4: "Notably, annotations coming from the Reference Genome Project (The Reference Genome Group of the Gene Ontology Consortium, 2009) are not identified so we were unable to establish any specific impact this may have had on the events of early 2012 (Figure 2C)"<br>The Reference Genome Project annotations can be identified either by a Panther ID in the "With/From" field or by a PAINT_REF in the "Reference" field of the GO annotation files.<br><br>Response: We greatly appreciate this tip as we are not sure where this is documented besides this reviewer's comment. Based on this, a quick analysis suggests that the Reference Genome Project is not the source of the early 2012 jump we noted, so we have removed mention of it from the manuscript.<br><br>Reviewer: 5. Page 10 Line 7: Figure 2C should be Figure 3C.<br><br>Response: We have corrected this error.<br><br>Reviewer: 6. Under "Global Trends" on the website, the Size of Gene Ontology graph appears to include obsolete terms (47204 in June 2018, compared with the total count of GO terms on the AmiGO website; currently 44947 non-obsolete). I was expecting to see a count of active terms, therefore it should be clarified what this graph is showing.<br>Good point. We have changed it so that for that plot, only non-obsolete terms are counted and this is indicated in the caption.<br><br>Reviewer #2: The use of Gene Ontology enrichment is widespread across biological sciences to investigate function or processes associated with particular conditions. This manuscript describes a resource called GOTrack that tracks changes in the Gene Ontology between releases over a number of years and enables users to investigate how the results of their enrichment analysis alter depending on the dataset that is used. This appears to be a resource that will be useful to many, including those working regularly with GO/GOA (e.g. those developing methods including for protein function prediction) and also more broadly researchers who want to identify the changes that occur in their system when they have different settings/conditions.<br><br>Response: We thank the reviewer for the positive comments.<br><br>Reviewer: The manuscript shows that annotations can vary considerably over time and further that the results of enrichment analyses can also change as a result. This appears to be interesting work but I also wonder if some of it is simply obvious. It is well established that Gene Ontology annotations are far from complete - I think less than 1% of proteins in uniprot have been assigned annotations with experimental evidence codes. Clearly there is much that is not known about protein function and therefore the Gene Ontology would be expected to develop over time. Some of this comes across in the discussion but I feel it would be better if the scene was set in the introduction.<br><br>Response: While it is obvious to us and the reviewers that GO is incomplete and changes, and we cited the previous literature on the topic, the reviewer might be surprised at how many biologists don't know, and Reviewer 1 (a GO insider) concurs that this needs wider attention. But our main point isn't that GO changes over time, but the difficulty in evaluating the changes and their impact. We provide a means to do so as well as a new evaluation of the impact of changes. To help make this clearer we have slightly reorganized and rewritten the first paragraphs of the introduction.<br><br>Reviewer: The manuscript shows that the GOTrack tool can be used to assess if enrichment analyses are stable over time - surely what researchers are interested in is whether the enrichment analysis that they perform is correct with the latest data set (which would be expected to contain the latest annotations)? It strikes me that if many new annotations are added in a particular release that use of previous release data may give different results and I would want to know if I can be confident based on the

	data that has been used rather than older data. It also seems that it is one of those problems where there will never be a correct answer unless all genes are fully annotated and this is unlikely to happen.<br><br>Response: The reviewer is right, that there will never be a correct answer, and we believe our work will help others realize this and use enrichment accordingly as an exploratory method.<br><br>Reviewer: The manuscript states that it is difficult to predict which GO terms will remain enriched in future datasets and this seems to be me to the most relevant or useful potential feature - looking to older datasets is ok but how useful is this?<br><br>Response: We concede that it remains to be seen how valuable the GOtrack tool proves to be, but we don't believe having predictions of future changes in GO/GOA are necessary for the enrichment tracking tool to have utility (and the reviewer's opening remarks seem to concur). Use cases (described in the manuscript) include checking the validity of past results, or to bolster confidence in new results (that they aren't merely a fluke of today's GO annotations).<br><br>Reviewer: In one of the examples (for ACTC1) the manuscript refers to IEA annotation - Inferred by electronic annotation. My understanding is that these are low confidence and variable should these really be used?<br><br>Response: We agree that it would be useful to be able to better separate annotations by evidence code and this is high on the list of features for a future release. In any case, other work has indicated that IEA annotations are not necessarily less reliable (Skunca et al., https://www.ncbi.nlm.nih.gov/pubmed/22693439). Whether they should be used or not may be a matter of opinion, but the fact is that many people do use IEA annotations.
**Additional Information:**	
**Question**	**Response**
Are you submitting this manuscript to a special series or article collection?	No
**Experimental design and statistics**<br><br>Full details of the experimental design and statistical methods used should be given in the Methods section, as detailed in our Minimum Standards Reporting Checklist. Information essential to interpreting the data presented should be made available in the figure legends.<br><br>Have you included all the information requested in your manuscript?	Yes
**Resources**<br><br>A description of all resources used, including antibodies, cell lines, animals and software tools, with enough information to allow them to be uniquely	Yes

identified, should be included in the Methods section. Authors are strongly encouraged to cite Research Resource Identifiers (RRIDs) for antibodies, model organisms and tools, where possible.<br><br>Have you included the information requested as detailed in our Minimum Standards Reporting Checklist?	
**Availability of data and materials**<br><br>All datasets and code on which the conclusions of the paper rely must be either included in your submission or deposited in publicly available repositories (where available and ethically appropriate), referencing such data using a unique identifier in the references and in the "Availability of Data and Materials" section of your manuscript.<br><br>Have you have met the above requirement as detailed in our Minimum Standards Reporting Checklist?	Yes

Manuscript
Click here to access/download;Manuscript;GOTrack_5_8_18.docx
Click here to view linked References

Monitoring changes in the Gene Ontology and their impact on genomic data analysis

Matthew Jacobson[1,2], Adriana Estela Sedeño-Cortés[3§], Paul Pavlidis[1,2]*

[1]Michael Smith Laboratories, University of British Columbia
[2]Department of Psychiatry, University of British Columbia
[3]Graduate Program in Bioinformatics, University of British Columbia
[§]Current address: Allen Institute for Brain Science, Seattle, Washington, USA

*Corresponding author:
177 Michael Smith Laboratories
2185 East Mall
University of British Columbia
Vancouver BC V6T1Z4, Canada
604 827 4157
paul@msl.ubc.ca

Running title: GOtrack

MJ: mjacobson@msl.ubc.ca

AES-C: adrianas@alleninstitute.org

PP: paul@msl.ubc.ca

Abstract

Background: The Gene Ontology (GO) is one of the most widely used resources in molecular and cellular biology, largely through the use of "enrichment analysis". To facilitate informed use of GO, we present GOtrack (https://gotrack.msl.ubc.ca), which provides access to historical records and trends in the Gene Ontology and GO annotations (GOA).

Findings: GOtrack gives users access to gene- and term-level information on annotations for nine model organisms as well as an interactive tool that measures the stability of enrichment results over time for user-provided "hit lists" of genes. To document the effects of GO evolution on enrichment, we analyzed over 2500 published hit lists of human genes (most over 9 years old). 53% of hit lists were considered to yield significantly stable enrichment results.

Conclusions: Because stability is far from assured for any individual hit list, GOtrack can lead to more informed and cautious application of GO to genomics research.

Keywords: Genetics, genomics, ontologies, gene function, bioinformatics

Background

The Gene Ontology (GO) has been widely adopted by computational and experimental biologists and Gene Ontology annotation (GOA) of genes is one of the most prominent descriptive features of major genome databases. The original paper describing GO [1] is among the most cited papers in the biomedical literature (over 14,000 citations, Clarviate Analytics Web of Science, accessed 1/2018). The popularity of GO is in large part due to the challenge of interpreting data generated from high-throughput technologies such as gene expression profiling.

In a typical simple setting, researchers contrast a genome-wide feature (e.g., gene expression levels or genetic association) in two experimental conditions and generate a list of genes, either ranked across the whole genome, or in the form of a "hit list" of selected candidates. Another way such lists can be generated is by clustering, such

as using protein interaction networks or coexpression; or by selecting genes harboring potentially pathogenic variants in cohort-based genome sequencing. To help extract biological meaning from those rankings and hit lists, it is now standard practice to use GO annotations in an “enrichment” framework.

The widespread use of these methods suggests it is important that users understand their underpinnings. However, despite the importance of GO, many users likely have little understanding of how it is developed, despite some effort on the part of the GO Consortium (GOC) to disseminate such information [2–4]. An important feature of GO is that it changes over time, as curation is performed. This has potentially major implications for the utility and interpretation of GO/GOA, but there is currently no means for users of GO to easily see this for themselves. Our goal in this paper is to help fill this gap and provide some insight into the actual impact of changes on data analysis.

The structure, content and curation of GO/GOA is the essential backdrop for the work we present so we review it briefly. It is important to distinguish the GO itself (the ontology) from the annotations (GOA), which connect genes to terms in GO. GO is organized into three sub-ontologies, representing Biological Processes, Molecular Functions and Cellular Components. Collectively these currently encompass over 40,000 concepts, arranged in a directed acyclic graph (like a tree, but with the potential for multiple paths from any leaf to the root).

Curation is managed through the Gene Ontology Consortium, in which member organizations such as model organism database curation teams provide annotations to a central repository. Genes may be associated with terms in the ontology using either manual curation (associated with a specific reference to the literature or based on a computational analysis reviewed by a curator) or “automatic” annotations that are not reviewed by curators. The different types of associations are represented by evidence codes, for example the automatic annotations receive the code “IEA” (“Inferred from electronic annotation”).

Annotations created by the curation process are referred to as “direct annotations” because they explicitly associate a GO term with a gene. Genes are also associated with terms indirectly via the graph structure of GO, referred to as inference. Thus, a gene that is directly annotated with the term “protein tyrosine kinase” is also implicitly annotated with the term “protein kinase” because that term is a parent term of “protein tyrosine kinase”. When the operation of propagating direct annotations through the GO hierarchies is completed (“transitive

closure” in graph theory terminology), the number of annotations available greatly increases, albeit at a range of granularities. These “indirect annotations” (also referred to as “inferred” or “propagated”) are as valid as direct annotations because GO enforces a “true path” rule [5]. In most analyses, it is important to use propagated annotations (the combination of direct and inferred annotations) [6].

Assessments of GO/GOA have recently turned to considerations of changes over time. For example, we quantified the effect that annotation have on the apparent (annotated) function of genes, showing that on average changes over short periods (months) are minor, but changes over longer periods are much more substantial [7]. This and other work has shown that GO enrichment results may not be stable over time. However, the effects of changes are not likely to be uniform across data sets nor easily predictable. Indeed, previous studies have been either anecdotal (considering a single or just a few examples [8–11]), with the largest study analyzing around 100 [12], or yielded mixed findings. Groß et al. (2012) found that enrichment results were stable based on analysis of two hit lists. Alam-Faruqe et al. considered changes in results to be improvements due to focused curation, based on analysis of two data sets. Others have emphasized instability [11,12] or reported mixed impacts [9]. Given this variety of results and interpretations, there is clearly a need for researchers to assess the stability of their own specific enrichment results.

Here we report the development and application of a database (GOtrack) that contains historical information on GO going back to the early 2000s for human and major model organisms. The GOtrack web site enables quick exploration of GO and GO annotations over time, and evaluation of how changes impact interpretation of analyses derived from GOA. Using the data in GOtrack, we present several analyses of trends in GO annotations, complementing earlier work. We performed a large-scale analysis of enrichment analysis results over time, using a large corpus of over 2500 “hit lists”. We confirm that GO enrichment analysis results can change over time. However, many were stable by objective measures even over time spans of greater than 10 years. It is our hope that GOtrack will enable more critical use of GO by biologists and computational researchers.

Findings

Construction and overview of GOtrack

We used data representing ontologies and annotations for nine organisms, dating as far back as 2001. Annotation data were not available for all organisms for all dates, with complete data for all nine organisms from November 15 2011 onwards. In total the data encompasses 206 monthly versions of GO and 1545 species-specific monthly editions of GOA yielding a grand total of 206,894,446 GO annotations (as of January 2018). Our overall procedures are outlined in Figure 1 (see Methods and further information is available on the GOtrack web site). The resulting database is complex and rich, with extensive information available at the gene or GO term level. While the web interface is the most complete and detailed way to interact with the data, we also offer a RESTful API to enable programmatic access to the data. Via this API, users can download GO annotations for a taxon, as well as GO, for any selected point in time. GOtrack does not contain all information on GO/GOA and thus complements other resources such as QuickGO [13] and AmiGO [14].

The GOtrack web interface offers views of history at the gene level, and at the GO term level. A third view provides a “global overview” of trends according to a variety of statistics. Finally, we offer a web tool to track changes in GO enrichment results over time. In this paper we provide only a high-level overview of basic functionality and readers are invited to explore the web interface for more information.

Figure 2A shows an example of one type of data offered in the gene view, for the human gene GRIN1 (glutamate ionotropic receptor NMDA type subunit 1; [15] and supplementary Figure 1A). The plot shows the number of GO terms directly annotated to the gene, with the mean of all genes from the same organism plotted for comparison. GRIN1 is consistently more highly annotated than the average, and its trajectory is typical in that annotations rise over time, interrupted by drops and recoveries. In general, such changes can be due to either annotation curation – addition or removal of terms annotated to the genes – or changes in the structure or content of the GO itself such as addition of terms or relations. The GOtrack interface also allows users to inspect changes in the use of evidence codes used to support an annotation, and directly compare annotations for a gene at up to four time points.

To help users interpret the changes in number of terms over time, we provide additional plots of statistics derived from the annotations. The first of these is of multifunctionality [16,17], which is related to the number of terms annotated to a gene, with a weighting to account for term specificity (where specificity is defined by how many genes are annotated with the term; see [17] for details). This more precisely captures how heavily annotated the gene is relative to other genes. The second derived statistic is semantic similarity. As time passes, changes in annotations can cause a gene to change "functional identity" [7]. To quantify this effect, we plot the Jaccard index between the annotations in the current edition to each previous edition. These and other plots and tables are presented on the web page for each gene.

The term-level view provides information on how a GO term has changed over time. This includes how many genes were annotated to it either in total (Figure 2B) or broken down by evidence type (for example [18] and Supplementary Figure 1B) as well as changes in the GO structure that impact the terms relationships. Finally, the Global Trends page [19] shows species-level summaries of the numbers of annotated genes, genes annotated per term, annotations per gene, and the size of GO itself.

Long-term trends in GOA

In this section we present some analysis of the data in GOtrack, focusing on annotations (rather than GO itself). As noted, genes vary strongly in how highly annotated they are, due to varying degrees of experimental and curation attention paid to the gene as well as potentially true biological differences in multifunctionality [17]. We previously reported that this bias tends to persist, that is, genes which are relatively highly annotated tend to stay that way [7]. We confirmed this is still the case five years later. For example, if we rank genes by how many direct annotations they have, the ranking at the earliest time point is correlated with the ranking at the latest time point (human: Spearman rank correlation 0.52; mouse 0.43; Arabidopsis 0.53). Thus we confirm that genes are not just unequal in their annotation, but that this inequality is stable over long periods.

The jumps seen in individual genes (e.g. Figure 2A) are not all independent events, as the course of the species-wide averages also has discontinuities (Figure 2A, grey). This is also apparent in a principal components analysis of the direct count matrix (Supplementary results, Supplementary Figure 2) We investigated this more completely in all nine GOtrack organisms at the level of total gene coverage (Figure 3A), genes annotated per term (Figure

3B), direct annotations per gene (Figure 3C) and inferred annotations per gene (Figure 3D). This reveals that large jumps and drops are sometimes simultaneously observed in multiple, or even all species. One such notable event was a rapid increase in the number of annotated genes starting March 2011 for Arabidopsis, mouse and zebrafish (Figure 3A). Another dramatic event was a large drop in the mean number of direct annotations per gene in March 2012 for all species (Figure 3C). The jump is not visible in the plots for indirect annotations (Figure 3D). This would be consistent with a large-scale purging of redundant annotations (rejecting higher-level terms that are inferable from more specific terms). Other jumps are species-specific, such as the large increase in Arabidopsis genes annotated per term in early 2012, followed by a large drop in late 2015 (Figure 3B).

At the gene level, large shifts in the numbers of annotations can be due to removal and replacement of annotations for the same term – a phenomenon we call “annotation churn”. For example, for the human gene ACTC1 [20], there is a pronounced rise in annotations in mid-2007, with a one-month dip in May 2008 (see screenshots in Supplementary Figure 3). GOtrack makes it easy to drill down into details. By examining the tabular results (Supplementary Figure 3A), it is found that one of the terms that was deleted during the dip was “apoptosis” (GO:0006915). Viewing the annotation history for that term on the gene, we see that the term was repeatedly added and removed (in 2007-2008), with the evidence code “IEA”. In June 2008 the term was annotated to the gene with a higher-grade curator-reviewed evidence code (ISS), where it remained (the term was also renamed to “apoptotic process”) – until it was removed again in December 2017 (Supplementary Figure 3B).

Tracking enrichment results

In addition to the exploratory aspects described so far, the other major component of the GOtrack system is an analysis tool which performs enrichment analysis at multiple time points ([21]; Supplementary Figure 4). The key idea is to observe whether an enrichment result is stable relative to a given point in time. The main input provided by the user is a “hit list” of genes. The output includes plots and detailed tables to help interpret the results and judge whether the results change over time. This includes direct comparisons of “before and after” sets of enriched terms. The measures we use for this comparison are discussed in the next section and in Methods. In addition to these statistics that summarize the overall stability of the results, the web interface provides term-level

stability measures. This makes it easy to see whether a term has been consistently "significant" over past editions.

The enrichment tool has some limitations: we use a simple over-representation method (as do many tools including the popular DAVID [22], and the "background" set of genes is not settable by the user: it is the set of all genes annotated at the particular time point. But because GOtrack provides downloads of GO and GOA for any date, users can confirm findings with software of their choice, provided it allows user-provided GO and GOA as inputs (such as ErmineJ, [16], whose annotation input format is directly supported).

Evaluating the stability of enrichment results

We hypothesized that changes in GO/GOA over time could cause changes in enrichment results to such an extent that they would be effectively unrecognizable and lead to a different interpretation of the results; as described in the introduction, previous studies of this question yielded somewhat mixed results on small numbers of test hit lists. In our approach to this question we used a corpus of gene lists from the Molecular Signatures Database (MSigDB) [23]. These are divided into two groups (after filtering, see Methods): 1327 curated "canonical pathways" (CP) and 2573 "chemical and genetic perturbations" (CGP). The latter correspond to published hit lists of the type usually investigated with enrichment analysis. We took advantage of the fact that each CGP hit list is associated with a publication, allowing the opportunity to see if the enrichment results obtained around the time of publication would have changed in the interim. We predicted the CP lists of established pathways would be more stable compared to the experimental CGP hit lists. The limitation of the MSigDB corpus is most of the publications are not very recent (median 11 years; range 0.4-16, 90% are >9.2 years old) and we have done little investigation of short-term stability.

For each hit list or pathway, we compute results of an enrichment analysis as it would have appeared at the GO/GOA edition nearest to the source publication date (see Methods for details). We then repeated the enrichment analysis using the most current GO/GOA edition (January 2018). This results in a range of timespans to have passed following publication. For the CP set, which do not all have an associated date, we computed results for the most recent GO/GOA edition and the earliest date available (January 2001). We used this extreme

date for comparison because we expected the CP set to have greater stability, so comparing to the earliest date is the “worst case scenario” for comparing to the experimentally-derived CGP sets.

Our first key observation is that on average for the CGP hit lists, the number of significant terms goes up dramatically (from 21±32 terms to 110±136 terms, mean ± standard deviation; $p<10^{-15}$, Wilcoxon rank sum test). The values are highly correlated (Figure 4A): hit lists that had few significant terms at the time of publication (henceforth t_0) had relatively few at the most recent timepoint (t_{now}) (rank correlation 0.54). These results also held for the Canonical Pathways (growing from 37±59 to 246±216 terms, correlation 0.57). It is likely that these increases are not just due to increased annotation, but the growth of GO to over 47,000 terms of increasing granularity.

The explosion in the number of significant terms is an obvious form of instability, but of course what matters more is whether the enriched terms resemble each other at t_{now} compared to t_0. To evaluate this, we did direct comparisons of the enriched terms associated with each hit list (at t_0 and t_{now}), using the Jaccard index (see Methods and Supplement). The Jaccard index was calibrated using a null distribution created by comparing pairs of unrelated hit lists (see Methods). To simplify the analysis, we binned the CGP hit lists by age into three groups of similar numbers of hit lists: up to 10 years, 10-12 years, and 12-16 years.

The results are shown in Figure 4B. Overall, 53% of the CGP hit lists had results which were more similar than 95% of the null trials. This fraction is much higher for relatively recent lists (71%, N=640) and lower for the older lists (55% for the middle tranche, N=960; and 38% for the oldest, N=973; Figure 4B). In comparison 75% of the Canonical Pathways remained above this threshold, despite most of the comparisons being done to the earliest possible time point. The overall rank correlation (unbinned) between stability and age is -0.34 (CGP; -0.39 for Canonical Pathways). This demonstrates that it is possible for results to maintain a substantial degree of similarity over periods of greater than 15 years, but that in general, drift in the semantic content of enrichment results is very substantial after 12-16 years and is substantial but less striking at shorter time spans (<10 years). In the Supplement we present examples of hit lists yielding high or low stability (Supplementary Results and Supplementary files).

A notable feature of the data shown in Figure 4B is that very low values of the complete Jaccard index were statistically significant. This shows the importance of using a null distribution to calibrate the scores, but clearly leaves something to be desired as a Jaccard index of 0.01 seems negligible. However, this effect is due in large part to the increase in the number of terms over time (Figure 4A), guaranteeing that the Jaccard index will drop. In attempts to explore this further, we tested six variants on the Jaccard index (see Supplement). While some of the alternatives have scales that are more intuitively matching expectations of what “stable” would represent on a scale of 0-1 (e.g., with 95%ile of the null equal to 0.41), the findings are qualitatively similar to the complete Jaccard (data for two additional measures are shown in Supplementary figure 5). Several of these alternative measures are implemented on the GOtrack web site. These measures are discussed further in the Supplement in the context of examples, along with discussion of the subjective nature of comparing enrichment results in an exploratory analysis.

We looked for factors that might contribute to stability. For the CGP hitlists, the number of genes in a hit list was not strongly predictive of Jaccard stability (rank correlation 0.18). It was only modestly correlated with the mean number of directly annotated terms (-0.12) or mean multifunctionality of the genes in the hit list (-0.09). There were more obvious trends for the canonical pathways lists, which have higher stability than the CGP lists on average, despite the (artificially) long time passed between t_0 and t_{now} (over 12 years; Figure 4B). The number of direct annotations per CP is higher (36 vs. 25.4 for CGP). However, this does not appear to explain the overall higher stability of the CP lists, because we get the same result for the subset of CP that has <35 mean direct annotations (mean of 22.9; correlation is -0.48; overall correlation -0.46). Thus hit lists that have more highly annotated genes have a tendency to be less stable. But given these low correlations (-0.12 for the CGP set) and without further insight, it appears to be difficult to predict (even in hindsight) which hit lists will yield stable results.

Discussion

In this work we present GOtrack, which to our knowledge is the only resource available that allows easy access to historical data on GO/GOA, and the only that allows inspection of the effects of changes over time on enrichment

result stability. Our analyses further highlight the necessity for users of GO/GOA to be cautious in their interpretation of any GO annotation, and to temper whatever trust they have in GO enrichment results.

Our evaluation of the stability of enrichment results differs in several important ways from earlier efforts. First, we matched GO and GOA for each time point (rather than fixing either GO or GOA while varying the other), which we feel is more realistic. We also analyzed a much larger number of hit lists (>2500 vs. a maximum of ~100 [12]) and considered time of publication to ensure comparisons were also realistic. But perhaps most importantly, we used a null distribution to calibrate the similarity measures, providing improved objective measures of what qualifies as stability. Overall our results are more optimistic about stability than Tomczak et al. (2018). Regardless, we concur with previous reports that changes in GO/GOA can make a substantial difference in results, but because of the high degree of variability and difficulty in finding fully satisfying quantitative measures that are often interpreted subjectively (see Supplement for discussion), our recommendation is that users of GO should judge for themselves by using GOtrack. Researchers who are reporting enrichment analyses can check which terms have been stable (for example, over the last five years). This provides a principled way to help narrow down complex enrichment results, a problem that many users of enrichment analyses struggle with.

An obvious limitation is GOtrack cannot see into the future. While the stability of any particular GO enrichment result might be high or low when looking backwards in time, it is generally impossible to know whether it will remain to be stable because knowledge of biology as represented in GO/GOA is a work in progress. Indeed, we found it is difficult to predict which hit list will give stable results. The strongest clue we could identify is how well annotated the genes in the hit list are: hit lists with highly annotated genes (mean direct annotation count) tend to be less stable. We speculate that this is because highly annotated genes have more changes to their annotations, which can drive shifts in enrichment results, but we have yet to explore this further and in any case the relationship is not strong enough to be usefully predictive. In addition we did not assess other possible factors influencing stability such as evidence codes [24], a topic we leave for future research.

GOtrack currently has some limitations. The enrichment tool uses a simple method and does not implement algorithms to asses multifunctionality biases [16]. Our data on GO/GOA is not complete: we did not import all of the fields from GO annotations files, the most useful of which for analysis purposes might be the annotation

source. Finally, the recently added concept of annotation extensions [25], which provide context for an annotation (for example, a cell type) are not handled by GOtrack.

Conclusions

The evolving and incomplete nature of GO/GOA has always been inherent and is well understood by the GO community. But it is seemingly less appreciated more broadly. For example, the extremely popular enrichment tool DAVID (over 32000 citations as of May 2018 [26]) did not update its GO annotations for nearly seven years, an eon in GO history (and at this writing DAVID has not been updated for nearly two years [27]). We find it interesting that there wasn't a massive outcry in response to the use of such out-of-date GO annotations, suggesting either ignorance or apathy. While it might seem obvious that one would always want to use the latest GO annotations, this can be questioned. GO/GOA can change dramatically in a see-saw fashion over a period of months, suggesting that not all changes are improvements. Furthermore, we report a strong tendency for hit lists to yield ever more significant terms over time (Figure 4A), and it is not clear this comes with any increase in useful information. It could be that using GO/GOA from an earlier, simpler era might be beneficial for enrichment analyses (using a GO slim [28] may approximate this concept). While we may not be able to settle that question here, it is clear that whatever version of GO/GOA is used, it cannot be treated as a gold standard. Enrichment analysis should be considered exploratory, and never used as a primary finding [29]. Computational researchers should also be cautious in using GO/GOA as an optimization target when developing and evaluating algorithms, especially since changes over time are not the only concern [7,17].

GOtrack should be a valuable resource for biologists to gain a greater understanding of where GO annotations come from and how they change over time, as well as their impact on the major use case for GO/GOA, enrichment analysis. Our analysis of the data in GOtrack also revealed a number of interesting features, and it is likely that deeper analyses can be used to gain more insight into patterns of curation that might influence future efforts.

Methods

Gene Ontology: Historical Gene Ontology files were retrieved from [30], specifically: Dates between 2001-01-01 - 2004-03-01 were obtained from separate process.ontology.<date>.gz, function.ontology.<date>.gz, and component.ontology.<date>.gz files and subsequently combined. Dates between 2004-04-01 and 2006-10-01 were obtained from gene_ontology.obo.<date>.gz. Dates after 2006-10-01 were obtained from gene_ontology_edit.obo.<date>.gz. These files exclude relationships that cross the three GO aspects and we restrict our analysis to IS_A and PART_OF relationships only.

Gene Ontology annotations: Historical species-specific annotation files were retrieved from [31], specifically: Dates between 2001-11-02 and 2016-05-09 were obtained from gene_association.goa_<species>.<edition>.gz. Dates after 2016-05-09 were obtained from a combination of goa_<species>.gpi.<edition>.gz and goa_<species>.gpa.<edition>.gz files. Mapping of historical annotations to a release of the Gene Ontology was done by selecting the ontology with the closest release date before that of the annotation file. Annotations were propagated up the GO graph as per the "true path rule" [5]. To convert release editions to dates, prior to edition 135 (July 2014) the release number of the file is compared to the dates given on the GOA news site [32]. For edition 135 onwards we use the date provided in the files. We note that there are some gaps in the available data, especially at early time points. For example, we lack data for human for September and October 2002. In addition, the spacing of dates is not uniform; while the median inter-edition gap is 28 days, there are a few gaps that are smaller (minimum 13 days) or correspondingly larger (e.g. 40 days).

Mapping of gene identifiers over time: Gene product annotations are tracked historically using their associated UniProt accession number(s) [33]. Each gene product in UniProt has a unique primary accession, called the 'Primary (citable) accession number'. In addition to this, a gene product may also have secondary accession numbers which could have been created historically from merges and/or splits. During a merge, the first accession is retained as the primary while all others become secondary. During a split, a new primary accession is created for all products involved while their original accessions are retained as secondary. An accession is only deleted when its corresponding entry has been removed from UniProt. The mapping of primary to secondary

accessions is retrieved from [34]. This mapping allows us to find the current primary accession of a historical annotation.

Enrichment analysis: GOtrack implements over-representation analysis using the hypergeometric distribution [16]. The background is the set of all annotated genes (for the time point being analyzed). For analyses presented in the paper, terms with between 20 and 200 genes were included, and only Biological Process terms were considered. The false discovery rate was controlled at 5% using the method of Benjamini and Hochberg [35]. The GOtrack enrichment tool allows these parameters to be varied by the user.

Data analysis: Many of the analyses described are based on files available via the GOtrack web site [36] including the "summary" files by edition, terms and genes. Analyses were conducted with custom scripts written in R [37,38] and python. Correlations are Spearman Rank correlations except where indicated otherwise.

Analysis of MSigDB hit lists: The MSigDB C2 collection [23] was downloaded from [39]. This corpus is divided into a set of "canonical pathways" (CP) and "chemical and genetic perturbations" (CGP). For the CGP hit lists, the publication associated with each hit list was extracted, and the date of publication (t_0) was used to identify the nearest matching version of GO/GOA in GOtrack. Each hit list was analyzed for enrichment as described above, for t_0 and a recent comparison time point (January 2018, t_{now}). We analyzed 2573 CGP hit lists that yielded at least five significant terms at either (or both) t_0 and the comparison time point. CP lists (n=1327 after filtering) were treated the same way, except t_0 was fixed at Nov 21 2005 (the mean date for the CGP lists).

To compare two sets of enrichment results, we explored several measures (see Supplement) but focus on a standard Jaccard index: $|E0 \cap E1|/|E0 \cup E1|$, where E0 and E1 are the sets of all significantly enriched GO terms for the same input hit list at two time points ("complete Jaccard"). The primary alternative measure we examined was a modified Jaccard that examines only the top five terms plus their inferred parent terms ("top-term-parents Jaccard"), similar to the measure proposed by [40]. See the supplement for details and discussion.

To generate a null distribution, we compare enrichment results from pairs of randomly-selected hits lists (i.e., coming from different publications). Instead of comparing a hit list's results for t_0 to t_{now}, the data are permuted so t_0 of one hit list is compared to t_{now} for a randomly-selected hit list (with the same constraint that at least one of them must have 5 or more significant GO terms). We analyzed 1000 such permutations of the data and pooled

them to generate the null distribution. This is an appropriate null because if two enrichment results from the same experiment (at two different time points) are less similar than what would be expected for two randomly picked independent experiments, we can say that the enrichment results are no longer similar according to the measure. This null also inherently addresses the tendency of some GO terms to recur more frequently than others in independent enrichment analyses [16].

Implementation: GOtrack is implemented in Java and JavaScript, and uses the PrimeFaces framework, with a MySQL database. The open source Highcharts (highcharts.com) visualization library is used for plotting. The data in GOtrack are automatically updated monthly. Because of the lag in when data are available from GOC, data for a given date appears in GOtrack up to 2 months after the stamped date.

Availability of supporting source code and requirements

- **Project name:** GOtrack
- **Project home page:** https://gotrack.msl.ubc.ca/ [41]; source code at https://github.com/PavlidisLab/gotrack [42]. Code and all data files needed to reproduce the analyses presented are provided [43]. An executable workspace is also available [44].
- **Operating system(s):** The web application runs under Linux. The web application works with major desktop operating systems (MacOSX, Windows, Linux).
- **Programming languages:** Java 8, JavaScript, Python
- **Other requirements:** The web application works with major web browsers.
- **License**: The contents (images, text, data) of the GOtrack web site are released under the Creative Commons Commons BY-SA 2.0 license. The GOtrack source code is open under the Apache 2.0 license.
- **RRID:** SCR_016399

An archival copy of the code and supporting data are also available via the *GigaScience* repository GigaDB [45].

Declarations

List of abbreviations:

- ACTC1: Actin, Alpha, Cardiac Muscle 1
- CGP: chemical and genetic perturbations
- CP: canonical pathways
- GO: Gene Ontology
- GOA: Gene Ontology Annotations
- GOC: Gene Ontology Consortium

- GRIN1: glutamate ionotropic receptor NMDA type subunit 1

Competing interests

PP is a member of the Gene Ontology Consortium Scientific Advisory Board. No other competing interests are declared.

Funding

Supported by NIH grant MH111099, an NSERC Discovery Grant and a Canadian Foundation for Innovation infrastructure grant. AES-C was supported in part by a CIHR-funded training grant in Bioinformatics.

Authors' contributions

PP conceived of the project and provided oversight. AES-C and PP developed the original GOtrack web site concept. MJ implemented GOtrack based on a prototype developed by AES-C. PP, MJ and AES-C performed analyses and drafted the manuscript.

Acknowledgements

We thank the GO Consortium for their efforts in creating GO/GOA. We are also dependent on the work of UniProt for protein identifiers. We thank Pascale Gaudet and Jesse Gillis for discussion, members of the Pavlidis lab for discussion and comments on the draft manuscript, and Dmitry Vavilov for technical support.

Supplementary files

- Supplementary results and discussion
- Supplementary Figure 1: Screen shots of the gene and term views in GOtrack
- Supplementary Figure 2: Principal components analysis of the direct annotation count matrix
- Supplementary Figure 3: Screen shots showing annotation history tracking for a gene (annotation churn)
- Supplementary Figure 4: Enrichment web interface

- Supplementary Figure 5: Analysis of MSigDB lists using alternative similarity measures
- Supplementary Figure 6: Correlations of stability measures
- Additional Files 1-3: Microsoft Excel spreadsheets providing examples of CGP enrichment results discussed in the supplement. (APPEL_IMATINIB_RESPONSE.enrichment.xlsx, BENPORATH_ES_2.enrichment.xlsx, ONDER_CDH1_SIGNALING_VIA_CTNNB1.enrichment.xlsx):

References

1. Ashburner M, Ball CA, Blake JA, Botstein D, Butler H, Cherry JM, et al. Gene ontology: tool for the unification of biology. The Gene Ontology Consortium. Nat Genet. 2000;25:25–9.

2. Blake JA. Ten Quick Tips for Using the Gene Ontology. PLoS Comput Biol. 2013;9:e1003343.

3. Gaudet P, Dessimoz C. Gene Ontology: Pitfalls, Biases, and Remedies. Gene Ontol Handb [Internet]. Humana Press, New York, NY; 2017 [cited 2018 Apr 9]. p. 189–205. Available from: https://link.springer.com/protocol/10.1007/978-1-4939-3743-1_14

4. Huntley RP, Sawford T, Martin MJ, O'Donovan C. Understanding how and why the Gene Ontology and its annotations evolve: the GO within UniProt. GigaScience. 2014;3:4.

5. Consortium TGO. Creating the Gene Ontology Resource: Design and Implementation. Genome Res. 2001;11:1425–33.

6. Rhee SY, Wood V, Dolinski K, Draghici S. Use and misuse of the gene ontology annotations. Nat Rev Genet. 2008;9:509–15.

7. Gillis J, Pavlidis P. Assessing identity, redundancy and confounds in Gene Ontology annotations over time. Bioinforma Oxf Engl. 2013;29:476–82.

8. Alam-Faruque Y, Huntley RP, Khodiyar VK, Camon EB, Dimmer EC, Sawford T, et al. The Impact of Focused Gene Ontology Curation of Specific Mammalian Systems. PLoS ONE. 2011;6:e27541.

9. Clarke EL, Loguercio S, Good BM, Su AI. A task-based approach for Gene Ontology evaluation. J Biomed Semant. 2013;4:S4.

10. Groß A, Hartung M, Prüfer K, Kelso J, Rahm E. Impact of ontology evolution on functional analyses. Bioinformatics. 2012;28:2671–7.

11. Wadi L, Meyer M, Weiser J, Stein LD, Reimand J. Impact of outdated gene annotations on pathway enrichment analysis. Nat Methods. 2016;13:705–6.

12. Tomczak A, Mortensen JM, Winnenburg R, Liu C, Alessi DT, Swamy V, et al. Interpretation of biological experiments changes with evolution of the Gene Ontology and its annotations. Sci Rep. 2018;8:5115.

13. Binns D, Dimmer E, Huntley R, Barrell D, O'Donovan C, Apweiler R. QuickGO: a web-based tool for Gene Ontology searching. Bioinforma Oxf Engl. 2009;25:3045–6.

14. Carbon S, Ireland A, Mungall CJ, Shu S, Marshall B, Lewis S. AmiGO: online access to ontology and annotation data. Bioinformatics. 2009;25:288–9.

15. GOtrack record for GRIN1 [Internet]. [cited 2018 Jul 27]. Available from: https://gotrack.msl.ubc.ca/genes.xhtml?accession=Q05586

16. Ballouz S, Pavlidis P, Gillis J. Using predictive specificity to determine when gene set analysis is biologically meaningful. Nucleic Acids Res. 2016;gkw957.

17. Gillis J, Pavlidis P. The impact of multifunctional genes on "guilt by association" analysis. PloS One. 2011;6:e17258.

18. GOtrack record for GO:0008021 - synaptic vesicle [Internet]. 2018 [cited 2018 Jul 27]. Available from: https://gotrack.msl.ubc.ca/terms.xhtml?query=GO%3A0008021

19. GOtrack trends page [Internet]. 2018 [cited 2018 Jul 27]. Available from: https://gotrack.msl.ubc.ca/trends.xhtml

20. GOtrack record for ACTC1 [Internet]. 2018 [cited 2018 Jul 27]. Available from: https://gotrack.msl.ubc.ca/genes.xhtml?accession=P68032

21. GOtrack enrichment tracker [Internet]. 2018 [cited 2018 Jul 27]. Available from: https://gotrack.msl.ubc.ca/enrichment.xhtml

22. Huang DW, Sherman BT, Lempicki RA. Systematic and integrative analysis of large gene lists using DAVID bioinformatics resources. Nat Protoc. 2009;4:44–57.

23. Subramanian A, Tamayo P, Mootha VK, Mukherjee S, Ebert BL, Gillette MA, et al. Gene set enrichment analysis: A knowledge-based approach for interpreting genome-wide expression profiles. Proc Natl Acad Sci U S A. 2005;102:15545–50.

24. Yu G, Lu C, Wang J. NoGOA: predicting noisy GO annotations using evidences and sparse representation. BMC Bioinformatics. 2017;18:350.

25. Huntley RP, Harris MA, Alam-Faruque Y, Blake JA, Carbon S, Dietze H, et al. A method for increasing expressivity of Gene Ontology annotations using a compositional approach. BMC Bioinformatics. 2014;15:155.

26. DAVID web site [Internet]. [cited 2018 Jul 27]. Available from: https://david.ncifcrf.gov/

27. DAVID Release & Version Information [Internet]. [cited 2018 Jul 27]. Available from: https://david.ncifcrf.gov/content.jsp?file=release.html

28. GO Slim and Subset Guide [Internet]. [cited 2013 Apr 11]. Available from: http://www.geneontology.org/GO.slims.shtml

29. Sedeño-Cortés AE, Pavlidis P. Pitfalls in the application of gene-set analysis to genetics studies. Trends Genet TIG. 2014;30:513–4.

30. GO FTP site [Internet]. [cited 2018 Jul 27]. Available from: ftp://ftp.geneontology.org/go/ontology-archive/

31. GOA FTP site [Internet]. [cited 2018 Jul 27]. Available from: ftp://ftp.ebi.ac.uk/pub/databases/GO/goa/old/<species>/

32. UniProt-GOA news [Internet]. [cited 2018 Jul 27]. Available from: https://www.ebi.ac.uk/GOA/news

33. Bateman A, Martin MJ, O'Donovan C, Magrane M, Alpi E, Antunes R, et al. UniProt: the universal protein knowledgebase. Nucleic Acids Res. 2017;45:D158–69.

34. UniProt primary/secondary mappings [Internet]. [cited 2018 Jul 27]. Available from: ftp://ftp.uniprot.org/pub/databases/uniprot/knowledgebase/docs/sec_ac.txt.

35. Benjamini Y, Hochberg Y. Controlling the false discovery rate: a practical and powerful approach to multiple testing. J R Stat Soc Ser B Methodol. 1995;57:289–300.

36. GOtrack download page [Internet]. [cited 2018 Jul 27]. Available from: https://gotrack.msl.ubc.ca/downloads.xhtml

37. Team RC. R: A language and environment for statistical computing. R Found Stat Comput Vienna Austria [Internet]. 2016 [cited 2017 Jan 25]; Available from: https://www.R-project.org/

38. Wickham H. ggplot2: Elegant Graphics for Data Analysis [Internet]. New York: Springer-Verlag; 2009 [cited 2018 Jun 1]. Available from: //www.springer.com/gp/book/9780387981413

39. MSigDB gene sets [Internet]. [cited 2018 Jul 27]. Available from: http://www.broadinstitute.org/gsea/msigdb/genesets.jsp

40. Mistry M, Pavlidis P. Gene Ontology term overlap as a measure of gene functional similarity. BMC Bioinformatics. 2008;9:327.

41. GOtrack [Internet]. 2018 [cited 2018 Jul 27]. Available from: https://gotrack.msl.ubc.ca/

42. GOtrack Github repository [Internet]. 2018 [cited 2018 Jul 27]. Available from: https://github.com/PavlidisLab/gotrack

43. GOtrack Dataverse record [Internet]. [cited 2018 Jul 27]. Available from: http://hdl.handle.net/11272/10596

44. GOTrack Code Ocean capsule [Internet]. 2018 [cited 2018 Jul 27]. Available from: https://doi.org/10.24433/CO.c6f199f4-0398-4294-a637-adda29895037

45. Jacobson M, Sedeño-Cortés AE, Pavlidis P. Supporting data for "Monitoring changes in the Gene Ontology and their impact on genomic data analysis" GigaScience Database. 2018. http://dx.doi.org/10.5524/100488

Figure legends

Figure 1: Overview of approach in constructing GOtrack. GO terms and GO annotations were obtained, matched by date, and harmonization of gene identifiers. Precomputed summary and aggregate statistics supplement the fine-grained information stored in the databases.

Figure 2: Examples of information provided by GOtrack for genes and terms. A. Number of terms directly annotated to the human gene GRIN1. Large drops and rises are observed superimposed over a general gradual increase in annotation since 2002 (black). In this example the large shifts are not accompanied by corresponding shifts in the species average (grey). B. Number of human genes directly annotated with the term “synaptic vesicle” (GO:0008021) over time, again showing transient drops and rises. Data from GOtrack was replotted for presentation. For corresponding screenshots, see Supplementary Figure 1.

Figure 3. Trends in taxon-wide annotation statistics. A: Number of annotated genes. B: Mean annotations per term (inferred + direct). C: Mean number of direct annotations per gene. D: Mean number of inferred (including direct) annotations per gene. Times of prominent discontinuities affecting multiple species in A and C are marked by dashed grey lines in all four panels.

Figure 4. Stability analysis of 2573 published hit lists A. Change in number of significant GO terms. Each point is one CGP hit list. Points are jittered to reduce overplotting. B. Similarity of enrichment results, using the complete Jaccard index. The CGP hit lists are binned into most recent (orange), old (green) and oldest (blue). The distribution for the canonical pathways is in black. The blue vertical line indicates the 95%ile of the null.

Click here to access/download
Supplementary Material
giga-320861.pdf

Click here to access/download
Supplementary Material
supp-1.zip
