# Supplementary material for: Monitoring changes in the Gene Ontology and their impact on genomic data analysis
Source: Gigascience. 2018 Aug 9;7(8):giy103. doi: 10.1093/gigascience/giy103 (PMC6113503; doi:10.1093/gigascience/giy103)
Supplement: Response_to_Reviewer_Comments_Original_Submission.pdf [file giy103_response_to_reviewer_comments_original_submission.pdf]

Author's Response To Reviewer Comments

Close

Dear Dr. Zauner:

We greatly appreciate the positive response to the work and the opportunity to submit a revision.

As there were only minor suggestions, we have not made any major changes. We have hopefully addressed all of the formatting and resource registration requirements.We have also created a Code Ocean capsule for the main analyses. At this time, I do not have the GigaDB information to include in the manuscript as I have not yet been contacted by your data curators.

All of the reviewer comments are addressed in the point-by-point, below.

Thank you for your consideration,

Paul Pavlidis PhD

Reviewer reports:

Reviewer #1: The manuscript by Jacobson et al. describes the GOTrack web interface that can be used to monitor the changes in Gene Ontology and its associated annotations over time.This is a novel method that potentially will assist users of the GO and its annotations to interpret their observations and analyses of gene product datasets. The paper is well written and the standard of English is good. As expected from these authors, a good knowledge of the GO and how it is developed and curated is demonstrated.The authors highlight the importance of recognising that GO and its associated annotations are dynamic and therefore any analysis is likely to change over time, a concept which is sadly lacking in many papers that use GO for interpreting results. Additionally, the authors explain that GOTrack can be used, in combination with annotation dataset reports from UniProt-GOA, to focus in on the possible reason for these changes. Therefore, this paper should go a long way to help further educate the users of GO. It has previously been suggested that, when performing enrichment analysis, researchers should look for key, relevant GO terms that have been enriched for their dataset in more than one analysis tool, due to the large variability between tool outputs (https://www.ncbi.nlm.nih.gov/pmc/articles/PMC3235096/), therefore the ability to compare a dataset over time using GOTrack is a very useful addition for assisting researchers to better interpret their datasets in an informed manner. The authors describe the various uses of GOTrack with appropriate examples that are easy to understand and well illustrated with figures. They compare GOTrack with previous evaluations of enrichment result stability and describe the measures they have taken to improve on the earlier studies. They also assist the reader with traceability of datasets by providing the sources of all files they have used. I have only a few minor comments.

Response: Thank you for the supportive comments.

Reviewer: 1. GOTrack currently only enables tracking changes of annotations for protein-coding genes, could the authors indicate whether they have considered including the newer GO annotation datasets that have been made available, i.e. macromolecular complexes and non-coding RNAs? Although there are significantly fewer annotations for these entities, I am aware of no other tools that provide direct enrichment analysis for these gene products, so this would be a very useful addition to consider.

Response: We haven't but will consider it for a future release. We agree that these annotations are much less explored, which is likely partly a function of their smaller number. As they increase there will be more impetus for us and others to build tools that support them.

Reviewer: 2. In Supp. Fig. 3A the authors show a comparison of 4 editions, but on the website it explains only how to compare two. I discovered by trial and error that you can add more editions by using + + , so this explanation should be added to the website.

Response: It is documented in the "?" tooltip in the upper left-hand corner of the plot. But your difficulty in noticing this led us to change the text to document the command-shift-click combination by default.

Reviewer: 3. Page 9 Line 21: remove 1st "the" from "because the we get the same result"

Response: We have corrected this error.

Reviewer: 4. Page 10 Line 4: "Notably, annotations coming from the Reference Genome Project (The Reference Genome Group of the Gene Ontology Consortium, 2009) are not identified so we were unable to establish any specific impact this may have had on the events of early 2012 (Figure 2C)"
The Reference Genome Project annotations can be identified either by a Panther ID in the "With/From" field or by a PAINT_REF in the "Reference" field of the GO annotation files.

Response: We greatly appreciate this tip as we are not sure where this is documented besides this reviewer's comment. Based on this, a quick analysis suggests that the Reference Genome Project is not the source of the early 2012 jump we noted, so we have removed mention of it from the manuscript.

Reviewer: 5. Page 10 Line 7: Figure 2C should be Figure 3C.

Response: We have corrected this error.

Reviewer: 6. Under "Global Trends" on the website, the Size of Gene Ontology graph appears to include obsolete terms (47204 in June 2018, compared with the total count of GO terms on the AmiGO website; currently 44947 non-obsolete). I was expecting to see a count of active terms, therefore it should be clarified what this graph is showing.
Good point. We have changed it so that for that plot, only non-obsolete terms are counted and this is indicated in the caption.

Reviewer #2: The use of Gene Ontology enrichment is widespread across biological sciences to investigate function or processes associated with particular conditions. This manuscript describes a resource called GOTrack that tracks changes in the Gene Ontology

between releases over a number of years and enables users to investigate how the results of their enrichment analysis alter depending on the dataset that is used. This appears to be a resource that will be useful to many, including those working regularly with GO/GOA (e.g. those developing methods including for protein function prediction) and also more broadly researchers who want to identify the changes that occur in their system when they have different settings/conditions.

Response: We thank the reviewer for the positive comments.

Reviewer: The manuscript shows that annotations can vary considerably over time and further that the results of enrichment analyses can also change as a result. This appears to be interesting work but I also wonder if some of it is simply obvious. It is well established that Gene Ontology annotations are far from complete - I think less than 1% of proteins in uniprot have been assigned annotations with experimental evidence codes. Clearly there is much that is not known about protein function and therefore the Gene Ontology would be expected to develop over time. Some of this comes across in the discussion but I feel it would be better if the scene was set in the introduction.

Response: While it is obvious to us and the reviewers that GO is incomplete and changes, and we cited the previous literature on the topic, the reviewer might be surprised at how many biologists don't know, and Reviewer 1 (a GO insider) concurs that this needs wider attention. But our main point isn't that GO changes over time, but the difficulty in evaluating the changes and their impact. We provide a means to do so as well as a new evaluation of the impact of changes. To help make this clearer we have slightly reorganized and rewritten the first paragraphs of the introduction.

Reviewer: The manuscript shows that the GOTrack tool can be used to assess if enrichment analyses are stable over time - surely what researchers are interested in is whether the enrichment analysis that they perform is correct with the latest data set (which would be expected to contain the latest annotations)? It strikes me that if many new annotations are added in a particular release that use of previous release data may give different results and I would want to know if I can be confident based on the data that has been used rather than older data. It also seems that it is one of those problems where there will never be a correct answer unless all genes are fully annotated and this is unlikely to happen.

Response: The reviewer is right, that there will never be a correct answer, and we believe our work will help others realize this and use enrichment accordingly as an exploratory method.

Reviewer: The manuscript states that it is difficult to predict which GO terms will remain enriched in future datasets and this seems to be me to the most relevant or useful potential feature - looking to older datasets is ok but how useful is this?

Response: We concede that it remains to be seen how valuable the GOtrack tool proves to be, but we don't believe having predictions of future changes in GO/GOA are necessary for the enrichment tracking tool to have utility (and the reviewer's opening remarks seem to concur). Use cases (described in the manuscript) include checking the validity of past results, or to bolster confidence in new results (that they aren't merely a fluke of today's GO annotations).

Reviewer: In one of the examples (for ACTC1) the manuscript refers to IEA annotation - Inferred by electronic annotation. My understanding is that these are low confidence and variable should these really be used?

Response: We agree that it would be useful to be able to better separate annotations by evidence code and this is high on the list of features for a future release. In any case, other work has indicated that IEA annotations are not necessarily less reliable (Skunca et al., https://www.ncbi.nlm.nih.gov/pubmed/22693439). Whether they should be used or not may be a matter of opinion, but the fact is that many people do use IEA annotations.

Close
