# Supplementary material for: Monitoring changes in the Gene Ontology and their impact on genomic data analysis
Source: Gigascience. 2018 Aug 9;7(8):giy103. doi: 10.1093/gigascience/giy103 (PMC6113503; doi:10.1093/gigascience/giy103)
Supplement: Reviewer_1_Report_(Original_Submission) -- Rachael Huntley — 7/5/2018 Reviewed [file giy103_reviewer_1_report_(original_submission).pdf]

Reviewer Report

Title: Monitoring changes in the Gene Ontology and their impact on genomic data analysis

Version: Original Submission **Date:** 7/5/2018

Reviewer name: Rachael Huntley

Reviewer Comments to Author:

The manuscript by Jacobson et al. describes the GOTrack web interface that can be used to monitor the changes in Gene Ontology and its associated annotations over time.
This is a novel method that potentially will assist users of the GO and its annotations to interpret their observations and analyses of gene product datasets. The paper is well written and the standard of English is good. As expected from these authors, a good knowledge of the GO and how it is developed and curated is demonstrated.

The authors highlight the importance of recognising that GO and its associated annotations are dynamic and therefore any analysis is likely to change over time, a concept which is sadly lacking in many papers that use GO for interpreting results. Additionally, the authors explain that GOTrack can be used, in combination with annotation dataset reports from UniProt-GOA, to focus in on the possible reason for these changes. Therefore, this paper should go a long way to help further educate the users of GO.
It has previously been suggested that, when performing enrichment analysis, researchers should look for key, relevant GO terms that have been enriched for their dataset in more than one analysis tool, due to the large variability between tool outputs (https://www.ncbi.nlm.nih.gov/pmc/articles/PMC3235096/), therefore the ability to compare a dataset over time using GOTrack is a very useful addition for assisting researchers to better interpret their datasets in an informed manner.

The authors describe the various uses of GOTrack with appropriate examples that are easy to understand and well illustrated with figures. They compare GOTrack with previous evaluations of enrichment result stability and describe the measures they have taken to improve on the earlier studies. They also assist the reader with traceability of datasets by providing the sources of all files they have used.

I have only a few minor comments.

1. GOTrack currently only enables tracking changes of annotations for protein-coding genes, could the authors indicate whether they have considered including the newer GO annotation datasets that have been made available, i.e. macromolecular complexes and non-coding RNAs? Although there are significantly fewer annotations for these entities, I am aware of no other tools that provide direct enrichment analysis for these gene products, so this would be a very useful addition to consider.

2. In Supp. Fig. 3A the authors show a comparison of 4 editions, but on the website it explains only how to compare two. I discovered by trial and error that you can add more editions by using <shift> + <command> + <click>, so this explanation should be added to the website.

3. Page 9 Line 21: remove 1st "the" from "because the we get the same result"

4. Page 10 Line 4: "Notably, annotations coming from the Reference Genome Project (The Reference Genome Group of the Gene Ontology Consortium, 2009) are not identified so we were unable to establish any specific impact this may have had on the events of early 2012 (Figure 2C)"
The Reference Genome Project annotations can be identified either by a Panther ID in the "With/From" field or by a PAINT_REF in the "Reference" field of the GO annotation files.

5. Page 10 Line 7: Figure 2C should be Figure 3C.

6. Under "Global Trends" on the website, the Size of Gene Ontology graph appears to include obsolete terms (47204 in June 2018, compared with the total count of GO terms on the AmiGO website; currently 44947 non-obsolete). I was expecting to see a count of active terms, therefore it should be clarified what this graph is showing.

Level of Interest

Please indicate how interesting you found the manuscript: An article of importance in its field

Quality of Written English

Please indicate the quality of language in the manuscript: Acceptable

Declaration of Competing Interests

Please complete a declaration of competing interests, considering the following questions:

- Have you in the past five years received reimbursements, fees, funding, or salary from an organisation that may in any way gain or lose financially from the publication of this manuscript, either now or in the future?
- Do you hold any stocks or shares in an organisation that may in any way gain or lose financially from the publication of this manuscript, either now or in the future?
- Do you hold or are you currently applying for any patents relating to the content of the manuscript?
- Have you received reimbursements, fees, funding, or salary from an organization that holds or has applied for patents relating to the content of the manuscript?
- Do you have any other financial competing interests?
- Do you have any non-financial competing interests in relation to this paper?

If you can answer no to all of the above, write 'I declare that I have no competing interests' below. If your reply is yes to any, please give details below.

I declare that I have no competing interests

I agree to the open peer review policy of the journal. I understand that my name will be included on my report to the authors and, if the manuscript is accepted for publication, my named report including any attachments I upload will be posted on the website along with the authors' responses. I agree for my report to be made available under an Open Access Creative Commons CC-BY license (http://creativecommons.org/licenses/by/4.0/). I understand that any comments which I do not wish to be included in my named report can be included as confidential comments to the editors, which will not be published.

I agree to the open peer review policy of the journal

To further support our reviewers, we have joined with Publons, where you can gain additional credit to further highlight your hard work (see: https://publons.com/journal/530/gigascience). On publication of this paper, your review will be automatically added to Publons, you can then choose whether or not to claim your Publons credit. I understand this statement. yes
