# Supplementary material for: Monitoring changes in the Gene Ontology and their impact on genomic data analysis
Source: Gigascience. 2018 Aug 9;7(8):giy103. doi: 10.1093/gigascience/giy103 (PMC6113503; doi:10.1093/gigascience/giy103)
Supplement: Reviewer_2_Report_(Original_Submission) -- Mark Wass — 7/25/2018 Reviewed [file giy103_reviewer_2_report_(original_submission).pdf]

Reviewer Report

Title: **Monitoring changes in the Gene Ontology and their impact on genomic data analysis**

Version: **Original Submission** **Date:** 7/25/2018

Reviewer name: **Mark Wass**

Reviewer Comments to Author:

The use of Gene Ontology enrichment is widespread across biological sciences to investigate function or processes associated with particular conditions. This manuscript describes a resource called GOTrack that tracks changes in the Gene Ontology between releases over a number of years and enables users to investigate how the results of their enrichment analysis alter depending on the dataset that is used. This appears to be a resource that will be useful to many, including those working regularly with GO/GOA (e.g. those developing methods including for protein function prediction) and also more broadly researchers who want to identify the changes that occur in their system when they have different settings/conditions.

The manuscript shows that annotations can vary considerably over time and further that the results of enrichment analyses can also change as a result. This appears to be interesting work but I also wonder if some of it is simply obvious. It is well established that Gene Ontology annotations are far from complete - I think less than 1% of proteins in uniprot have been assigned annotations with experimental evidence codes. Clearly there is much that is not known about protein function and therefore the Gene Ontology would be expected to develop over time. Some of this comes across in the discussion but I feel it would be better if the scene was set in the introduction.

The manuscript shows that the GOTrack tool can be used to assess if enrichment analyses are stable over time - surely what researchers are interested in is whether the enrichment analysis that they perform is correct with the latest data set (which would be expected to contain the latest annotations)? It strikes me that if many new annotations are added in a particular release that use of previous release data may give different results and I would want to know if I can be confident based on the data that has been used rather than older data. It also seems that it is one of those problems where there will never be a correct answer unless all genes are fully annotated and this is unlikely to happen.

The manuscript states that it is difficult to predict which GO terms will remain enriched in future datasets and this seems to be me to the most relevant or useful potential feature - looking to older datasets is ok but how useful is this?

In one of the examples (for ACTC1) the manuscript refers to IEA annotation - Inferred by electronic annotation. My understanding is that these are low confidence and variable should these really be used?

Level of Interest

Please indicate how interesting you found the manuscript: Choose an item.

Quality of Written English

Please indicate the quality of language in the manuscript: Choose an item.

Declaration of Competing Interests

Please complete a declaration of competing interests, considering the following questions:

- Have you in the past five years received reimbursements, fees, funding, or salary from an organisation that may in any way gain or lose financially from the publication of this manuscript, either now or in the future?
- Do you hold any stocks or shares in an organisation that may in any way gain or lose financially from the publication of this manuscript, either now or in the future?
- Do you hold or are you currently applying for any patents relating to the content of the manuscript?
- Have you received reimbursements, fees, funding, or salary from an organization that holds or has applied for patents relating to the content of the manuscript?
- Do you have any other financial competing interests?
- Do you have any non-financial competing interests in relation to this paper?

If you can answer no to all of the above, write 'I declare that I have no competing interests' below. If your reply is yes to any, please give details below.

I declare that I have no competing interests.

I agree to the open peer review policy of the journal. I understand that my name will be included on my report to the authors and, if the manuscript is accepted for publication, my named report including any attachments I upload will be posted on the website along with the authors' responses. I agree for my report to be made available under an Open Access Creative Commons CC-BY license (http://creativecommons.org/licenses/by/4.0/). I understand that any comments which I do not wish to be included in my named report can be included as confidential comments to the editors, which will not be published.

I agree to the open peer review policy of the journal

To further support our reviewers, we have joined with Publons, where you can gain additional credit to further highlight your hard work (see: https://publons.com/journal/530/gigascience). On publication of this paper, your review will be automatically added to Publons, you can then choose whether or not to claim your Publons credit. I understand this statement. yes
